# Dementia with Lewy bodies and gait neural basis: a cross-sectional study

**DOI:** 10.1186/s13195-024-01539-z

**Published:** 2024-07-30

**Authors:** Adele Sainsily-Cesarus, Elise Schmitt, Lionel Landre, Anne Botzung, Lucie Rauch, Catherine Demuynck, Nathalie Philippi, Paulo Loureiro de Sousa, Catherine Mutter, Benjamin Cretin, Catherine Martin-Hunyadi, Frederic Blanc

**Affiliations:** 1 Geriatrics Department, University Hospital of Strasbourg, CM2R (Memory Resource and Research Centre), Strasbourg, France; 2 University of Strasbourg, CNRS, UMR 7357 and FMTS (Fédération de Médecine Translationnelle de Strasbourg), team IMIS, ICube laboratory, Strasbourg, France; 3 Faculty of Medicine, University of Strasbourg, Strasbourg, EA-3072 France; 4 University Hospital of Strasbourg, CIC INSERM 1434, Strasbourg, France

**Keywords:** Dementia with lewy bodies, Walking speed, Mid-cingulate cortex, Hippocampi, Magnetic resonance imaging, Voxel-based-morphometry, VBM

## Abstract

**Background:**

Dementia with Lewy Bodies (DLB) is responsible for cognitive-behavioural disorders but also for gait disorders. The latter are thought to be related to parkinsonism, but the neural bases of these disorders are not well known, especially in the early stages. The aim of this study was to investigate by volumetric Magnetic Resonance Imaging the neuronal basis of gait disorders in DLB patients, compared to Healthy Elderly Controls and Alzheimer’s Disease patients.

**Methods:**

Clinical examination with motor assessment including 10-meter walking speed, one-leg balance and Timed Up and Go test, a comprehensive neuropsychological evaluation and 3D brain Magnetic Resonance Imaging were performed on 84 DLB patients, 39 Alzheimer’s Disease patients and 22 Healthy Elderly Controls. We used Statistical Parametric Mapping 12 to perform a one-sample t-test to investigate the correlation between each gait score and gray matter volume (*P* ≤ 0.05 corrected for family-wise error).

**Results:**

We found a correlation for DLB patients between walking speed and gray matter decrease (*P* < 0.05, corrected for family-wise error) in caudate nuclei, anterior cingulate cortex, mid-cingulate cortex, hippocampi, supplementary motor area, right cerebellar cortex and left parietal operculum. We found no correlation with Timed Up and Go test and one-leg balance.

**Conclusion:**

Gait disorders are underpinned by certain classical regions such as the cerebellum and the supplementary motor area. Our results suggest there may be a motivational and emotional component of voluntary gait in DLB subjects, underpinned by the cingulate cortex, a spatial orientation component, underpinned by hippocampi and suggest the involvement of brain processing speed and parkinsonism, underpinned by the caudate nuclei.

**Trial registration:**

The study protocol has been registered on ClinicalTrials.gov. (NCT01876459) on June 12, 2013.

## Introduction

Alzheimer’s disease (AD) and dementia with Lewy Bodies (DLB) are the two main cognitive neurodegenerative disorders. AD is the most common of these disorders and accounts for 50–75% of dementia [1]. DLB represents 15–20% of the neurocognitive pathologies in older people [2], but its prevalence is likely underestimated due to misdiagnosis [3].

Major neurocognitive disorder or dementia is a major risk factor for falls: it multiplies this risk by more than 10 in people over 65 years old [4]. The annual incidence of falls has been estimated at 50% in patients with dementia, which may nevertheless be underestimated as patients often do not keep track of their falls [5]. These falls can be traumatic, leading to a loss of confidence and autonomy, and often cause significant psychological trauma, accelerating institutionalization and even increasing mortality rates [6].

Falls are twice as frequent for DLB patients: in one year, almost all DLB patients fell, compared to half of AD patients [4]. Some authors explain this increased risk of falls in DLB patients by the gait and balance disorders that appear to be more marked in DLB patients than in AD subjects [5, 7]. In fact, DLB subjects have an extrapyramidal syndrome (akinesia, rigidity) responsible for slower walking, with a marked variation in spatial and temporal parameters such as step length [7], as well as a significantly altered pace [8].

Hippocampal atrophy has long been linked to AD, but in recent years it has become apparent that this atrophy could be found in other types of dementia, including DLB, and is therefore sensitive to, but not specific to, AD [9]. The current literature proved hippocampal atrophy to cause memory disorders [10, 11] and also linked this volume loss to poor walking performance in individuals with mild cognitive impairment.

A meta-analysis using the voxel-based morphometry (VBM) method to assess atrophy in DLB patients highlighted atrophy in regions such as the insula, putamen and lateral temporal lobe [12]. The current literature focuses more on the neural basis of the brain regions involved in cognitive disorders caused by DLB, and places less emphasis on motor disorders, even though they lead to falls, including in the early stages of DLB [4]. Despite the limited literature, there appears to be a link between central gray nuclei involvement and parkinsonism [13].

The objective of this study was to investigate the neural basis of gait disorders in DLB patients, compared to healthy elderly subjects and AD patients, by using volumetric Magnetic Resonance Imaging (MRI). We hypothesized that walking disorders could be caused by the damage to the central gray nuclei associated with extrapyramidal syndrome and by the damage to frontal regions responsible for executive dysfunction.

## Population and methods

### Study population

The patients and Healthy Elderly Controls (HEC) included in this study were recruited from the Memory Resource and Research Center (CM2R) in Strasbourg, France, and were already enrolled in the AlphaLewyMA cohort (clinicaltrials.gov, NCT01876459). The initial objective of this cohort was to establish a differential diagnosis between DLB and AD based on the determination of alpha-synuclein in cerebrospinal fluid in patients with mild cognitive impairment or mild to moderate dementia [13]. Patients aged over 45 years were included in three centers: the Geriatric Day Hospital, the Neuropsychology Unit of the University Hospital of Strasbourg, and the Geriatric Day Hospital of the Hospital of Colmar.

On inclusion in the study, each participant underwent a brain MRI and a clinical examination, including an assessment of gait, an evaluation of autonomy and neuropsychological tests. The diagnosis of AD was based on the criteria of Dubois et al. of 2007 [10] and the diagnosis of DLB was based on the revised criteria of McKeith et al. of 2005 [14]. There was no restriction on inclusion regarding the use of medication.

Patients with severe dementia (Mini-Mental State Examination (MMSE) ≤ 9), with both diseases, with other types of dementia, other associated progressive comorbidities or vision or hearing impairments were not included [15].

### Clinical protocol

Participants with cognitive disorders were reassessed every six months while healthy elderly subjects had an annual follow-up.

At each visit, a complete neurological examination was performed, looking for parkinsonism using the UPDRS III (Unified Parkinson’s Disease Rating Scale [16]) reduced to four items: bradykinesia, rigidity, tremor and facial expressions for all participants.

A motor evaluation was performed, including walking speed (10-meter walk test) assessed in a standardised walking corridor [17], the one-leg balance test [18] and the Timed Up and Go test (TUG) [19]. One-leg balance was considered successful and was rated two if the subject could balance for five seconds on each foot; performance was rated one if the subject could balance on only one foot and zero if the subject could not balance on either foot.

Falls during the previous six months were noted by the patients themselves or their caregivers, but their exact frequency was not necessarily written down.

A complete neuropsychological evaluation including an MMSE was performed annually to avoid a retest effect. Autonomy was assessed using the Instrumental Activities of Daily Living (IADL) and Activities of Daily Living (ADL) scales. Cognitive fluctuations were assessed by the Mayo Clinic fluctuations scale.

### Study design

We decided to run a cross-sectional study and chose to perform one visit per subject. We then selected the most complete visit which included neurological examination, motor evaluation (walking speed, TUG and one-leg balance), brain MRI, MMSE and IADL.

### MRI acquisition

All the selected patients underwent multimodal brain MRI, performed using a Siemens Magnetom Verio 3 Tesla device with a 32-channel probe.

The high-resolution 3D T1-weighted anatomical sequence with an acquisition time of 7.38 min was our sequence of interest. We used the following parameters: an echo time of 2.53 ms, an inversion time of 900 ms, a rotation angle of 9°, a repetition time of 1900 ms and an isotropic voxel size of 1 mm^3^.

### Voxel-based-morphometry (VBM)

To explore the existence of correlations between the deficits observed in the different motor evaluation scores and the concentration of Gray Matter (GM), we used the VBM method implemented in SPM12 (Statistical Parametric Mapping 12) under Matlab R2017b (The MathWorks, Inc., Natick, Massachusetts, US). Between-group differences in GM volume were assessed using the SPM12. Age, sex, and total GM volume were included in the design matrix as covariates of no interest.

**Fig. 1:**
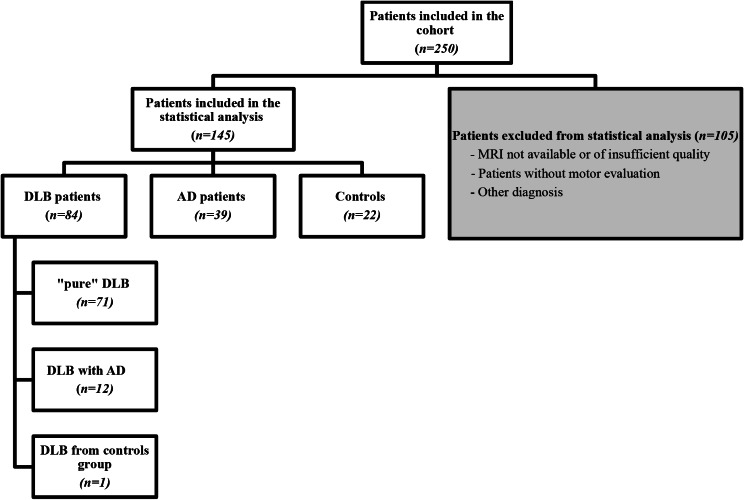
Flowchart of the study analyzing the neural basis of gait in dementia with Lewy bodies. DLB: Dementia with Lewy bodies, AD: Alzheimer’s Disease, MRI: Magnetic Resonance Imaging

Each MR image was visualized to judge its quality. Wherever possible, the MRI closest to the motor evaluation was selected for each patient. Due to movement artifacts that could prevent the interpretation of some MR images, we were sometimes forced to choose an image acquired at a date further away from the motor evaluation (without exceeding a two-year delay), or even to eliminate patients (see Fig. 1, flowchart).

Once the selection of the best MRI image was completed, we converted the images from Digital Imaging and Communications in Medicine to Neuroimaging Informatics Technology Initiative format. We used the pre-treatment and DARTEL (Diffeomorphic Anatomical Registration Through Exponentiated Lie algebra) stages. A spatial normalization according to the Montreal Neurological Institute space (MNI) was carried out allowing an affine modulation (translation, rotation, shear) to correct the various interindividual morphological variations. The images were then segmented into six classes, of which we selected two: gray and white matter. With these elements, we generated a DARTEL template specific to our population, by successive distortions of individual images. During this procedure, the individual GM images were modulated to preserve local volume and subjected to an 8 mm full width at half maximum Gaussian filtering. Before performing the statistical analyses, a visual check was conducted to ensure the quality of the images and the correct delimitation of white and gray matter, particularly in the lateral ventricles and the cortical ribbon.

Three parameters of motor evaluation were tested separately as regressor, with age, sex and total GM volume in covariates.

### Statistical analyses

For the analysis of population characteristics, we conducted t-tests or Fisher’s exact test. Linear regression analyses were performed to adjust walking speed for age and height using Jamovi^®^ software. Correlation analyses were performed using Spearman’s non-parametric test.

For the VBM analysis, intergroup differences were estimated with t-tests or ANOVAs, including age, sex and amount of total GM as covariates. An alpha threshold of 0.05 corrected FWE (Family-Wise Error rate) was applied at first. In the absence of significant results, the threshold was lowered by 0.001 uncorrected at the voxel level and then all results were corrected at the cluster level (*P* = 0.05 False Discovery Rates (FDR) corrected).

## Results

### Characteristics of the subjects

Twelve participants in the DLB group had associated AD (DLB-AD). These participants had the clinical features of DLB. Given that gait disorders are often more severe in DLB patients than in AD patients [4] and that the main aim of this study was to investigate the neural basis of gait disorders in DLB patients, it was coherent to keep them in the DLB group.

Demographic, cognitive and motor characteristics are detailed in Table 1.

**Table 1:** Comparison of demographic, cognitive and motor characteristics between DLB group, AD group and HEC group

Characteristics	DLB group N = 84	AD group N = 39	HEC group N = 22
Sex Female (ratio)	0.57^a, c^	0.59^a, b^	0.64 ^b, c^
Age, years (mean (SD))	70.6 (10.1)^c^	75.6 (7.6) ^a, b^	66.5 (8.7)^c^
Height, cm (mean (SD)) N	167 (8.8)	165 (10.6)	165 (8.9)
	N = 71/84	N = 29/39	N = 19/22
BMI, kg/m² (mean (SD)) N	27 (4.6)	27.5 (4.6)	25 (4.1)
	N = 71/84	N = 29/39	N = 19/22
IADL [0–4] (mean (SD))	3.2 (1)^a^	3.2 (1.1)^a^	4 (0)
Antiparkinsonian medication (ratio)	0.38 ^a, c^	0	0
Cholinesterase inhibitors (ratio)	0.44	0.43	0
Occasional fallers (ratio)	0.06	0.05	0.1
Recurrent fallers (ratio)	0.08^a^	0.025	0
**Cognitive variables**			
Education, years (mean (SD)) N	11.3 (4.2)^a^	11.8 (4.2)^a^	13.5 (2.1)
	N = 79/84	N = 35/39	N = 20/22
Laterality, right-handed (ratio)	0.89	0.82	0.95
MMSE [0–30] (mean (SD)) N	25.4 (3.9)^a, c^	23.5 (4.4)^a, b^	29.1 (0.9)^b, c^
	N = 84/84	N = 38/39	N = 22/22
Fluctuation score [0–4] (mean (SD))	1.4 (1.3)^a, c^	0.56 (1.2)^b^	0.27 (0.55)^b^
**Motor variables**			
Parkinsonism score [0–16] (mean (SD))	3.6 (1.7)^a, c^	1.2 (0.4) ^a, b^	1 (0) ^b, c^
Walking speed, m/sec (mean (SD))	0.87 (1.56)	0.84 (0.78)	1.04 (2.5)
Timed Up and Go test, sec (mean (SD))	12.5 (6.5)^a^	12.8 (14.5)^a^	7.8 (2.2)^b, c^
One-leg balance (ratio)	0.41^a^	0.44^a^	0.86 ^b, c^

DLB group, Dementia with Lewy Bodies group; AD group, Alzheimer’s Disease group; HEC group, Healthy Elderly Controls group; BMI, Body Mass Index; MMSE, Mini-Mental State Examination; SD Standard Deviation; N: number

^a^*P* < 0.05: versus HEC group

^b^*P* < 0.05: versus DLB group

^c^*P* < 0.05: versus AD group

Mean age was higher in the AD group when compared to the DLB group (*P* = 0.001) and the HEC group (*P*<0.001) (75.6 years, 70.6 years and 66.5 years respectively).

There was no significant difference in height or Body Mass Index (BMI) between the groups.

None of the participants in the HEC group were taking antiparkinsonian medication or cholinesterase inhibitors. As for anticholinesterase drugs, 43.5% of the AD group and 44% of the DLB group were taking them, with no statistically significant difference between the two groups (*P =* 0.943). For antiparkinsonian drugs, only participants in the DLB group benefited, for a total of 38%. The treatments were not interrupted during the research.

There were no significant differences between the groups regarding the occurrence of falls in the previous 6 months. On the other hand, analysis of the participants’ profile concerning the recurrence of falls showed that among the participants in the AD group, there were 2 occasional fallers (5%) and 1 recurrent faller (2.5%), in the DLB group, there were 5 occasional fallers (6%) and 7 recurrent fallers (8.3%) and in the HEC group, there were 2 occasional fallers (10%) and no recurrent fallers. The only significant difference between these three groups was a higher number of recurrent fallers in the DLB group than in the HEC group (*P* = 0.006).

Mean performance on the MMSE was poorest in the AD group, with a significant difference with the HEC group (*P* < 0.001) and even with the DLB group (*P* = 0.03).

Mean walking speed was not significantly different between the 3 groups, even when adjusted for age and height, variables known to modify walking speed (DLB group vs. HEC group, *P =* 0.876; AD group vs. HEC group, *P =* 0.187; DLB group vs. AD group *P =* 0.649).

Mean score of the TUG was significantly better in the HEC group when compared to the other two groups (*P* = 0.0001).

On the one-leg balance test, the HEC group had a success rate of 86.4%, which was significantly better than the other two groups (*P* < 0.0001).

We found no correlation between cognitive functions with the MMSE and gait speed, TUG or one-leg balance test in the HEC group. However, there was a correlation between the MMSE and the various gait parameters in the DLB group (gait speed *P* < 0.0001, TUG *P* < 0.0001 and one-leg balance test *P =* 0.004), and for the AD group, there was a correlation between the MMSE and the TUG (*P =* 0.04).

### VBM analysis in DLB group

#### Gray matter volumes

Overall GM volumes differed significantly in pairs between the HEC group (606 +/- 72 ml), the DLB group (554 +/- 82 ml) and the AD group (522 +/- 70 ml).

There was a correlation between total GM volume and cognitive assessment using the MMSE in the AD group (*P* = 0.0001) and the DLB group (*P* = 0.005), but not in the HEC group.

For gait parameters and total GM volume, there was a correlation only for gait speed in the HEC (*P* = 0.04) and DLB groups (*P* = 0.04), but not in the AD group; however, there was no correlation between GM volume and TUG or single-leg balance in any of the groups.

#### Walking speed

**Fig. 2:**
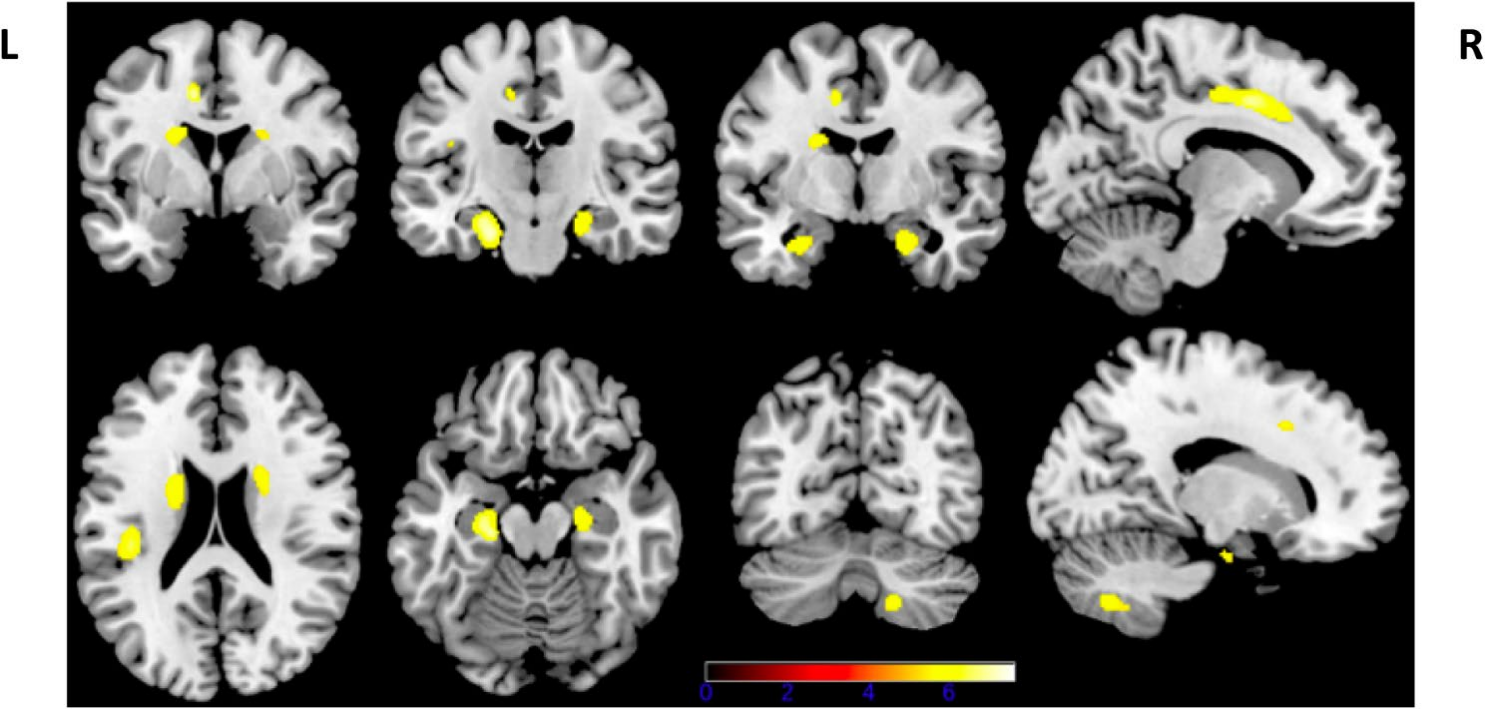
Gray matter concentration correlated to walking speed in dementia with Lewy bodies. Walking speed correlates with bilateral local decrease gray matter concentration in the caudate nuclei, hippocampi, anterior cingulate cortex, midcingulate cortex and supplementary motor area, as well as in the right cerebellar cortex and left parietal operculum (FWE corrected, *P* < 0.05). L: Left; R: Right

For an alpha threshold of 0.05 FWE corrected, we found a relationship between decrease walking speed and bilateral local decrease GM concentration in the caudate nuclei, hippocampi, anterior cingulate cortex (ACC), midcingulate cortex (MCC) and supplementary motor area (SMA), as well as in the right cerebellar cortex and left parietal operculum (PO) (Fig. 2).

The same regions, including the hippocampi, were also found for an alpha threshold of 0.05 FWE corrected when the 12 DLB-AD patients were removed from the analysis.

The walking speed results are summarized in Table 2.

**Table 2:** Walking speed results with standard covariates in dementia with Lewy bodies

Parameter tested	Regions	Laterality	Corrected threshold (FWE)	Cluster size (k)	T	MNI Coordinates (x, y, z in mm)
**Walking speed**	*Hippocampi*	Left	0.05	882	7.49	-21, -21, -21
		Right	0.05	514	6.58	21, -10, -24
	*Parietal operculum*	Left	0.05	265	6.57	-40, -27, 22
	*Anterior Cingulate Cortex*	Right	0.05	146	5.49	12, 27, 27
	*Midcingulate Cortex*	Right	0.05	146	5.75	15, 16, 38
		Left	0.05	505	6.89	-10, 0, 42
	*Supplementary Motor Area*	Left	0.05	505	6.07	-12, -14, 45
		Right	0.05	146	5.75	15, 16, 38
	*Caudate nuclei*	Left	0.05	251	6.05	-21, 0, 22
		Right	0.05	185	5.71	22, 9, 20
	*Cerebellum*	Right	0.05	181	5.42	18, -66, -45

The fluctuation score, the total parkinsonism score, the years of education or laterality had no significant effect on our results obtained with walking speed. However, with the covariate MMSE, hippocampi and midcingulate cortex were the only region correlated to walking speed (*P* = 0.05, FWE corrected).

#### One-leg balance

A correlation with GM concentration of caudate nuclei, midcingulate cortex and left supplementary motor area was found at the uncorrected threshold of *P* = 0.001 but did not persist after FWE and FDR correction at the cluster level.

#### Timed-Up and go test

No significant results were found at the voxel-wise threshold of 0.05 FWE for TUG. However, using a voxel-wise threshold of *P* = 0.001 uncorrected and then corrected at the cluster level at *P* = 0.05 FDR, we had significant results: the bilateral anterior- and midcingulate cortices, bilateral SMA, left parietal operculum and right cerebellar cortex.

By performing these same analyses with walking speed in AD patients, we found a correlation with the bilateral cerebellar cortex at an uncorrected voxel-wise threshold of *P* = 0.001 that did not persist after correction at the cluster level (FDR or FWE). We did not find any significant result with TUG and one-leg balance in the AD group. No significant correlations with GM using VBM were found in the HEC group.

Intergroup comparisons were made by adjusting for age, sex and total GM volume.

In the DLB group, compared with the HEC group, there was a decrease in GM concentration in the right temporal pole using a voxel-wise threshold of *P* = 0.001 uncorrected and then corrected at the cluster level at *P* = 0.05 FDR, without specifically identifying the regions found to be involved in gait disorders in the DLB group (hippocampi, parietal operculum, cingulate cortex, supplementary motor area, caudate nuclei and cerebellum).

Comparing the AD group with the HEC group, it appears that GM concentrations in the temporal poles, hippocampi and amygdala are lower in the AD group than in the HEC group using a voxel-wise threshold of *P* = 0.001 uncorrected and then corrected at the cluster level at *P* = 0.05 FDR. Again, the only correlation found for the AD group was between walking speed and cerebellar regions, a correlation that did not persist after correction at the cluster level, and cerebellar regions were not reduced in volume compared with the HEC group.

The AD group compared with the DLB group showed a loss in GM volume in the amygdala and in the anterior part of the hippocampi (*P* = 0.05, FWE corrected).

The correlations obtained for the DLB group are therefore not in atrophied areas and therefore appear to be specific to walking.

## Discussion

The aim of this study was to investigate which brain regions were involved in gait disorders of DLB patients compared to participants with AD or to HEC. Volumetric analyses revealed a correlation between reduced walking speed and gray matter decrease in regions expected to be related to motor function: caudate nuclei, cerebellum and SMA, but the left parietal operculum was also involved (proprioceptive integration). Furthermore, when all covariates were included in the analysis, and in particular the MMSE, the two brain regions most strongly correlated with reduced walking speed were in the limbic regions: cingulate cortex and hippocampi. No such correlation was found for the other two groups.

### Gait speed and cognition

There was a correlation between the MMSE and the various gait parameters in the DLB group, and for the AD group, there was a correlation between the MMSE and the TUG.

Few studies have looked specifically at the impact of cognition on gait parameters in DLB. Sverdrup et al. observed a decline in walking speed across the cognitive spectrum, starting with people with subjective cognitive decline [20]. Participants with mild cognitive impairment also showed a reduction in lower limbs muscle strength, balance and grip strength. Participants with dementia had significantly lower scores on all measures of physical performance. Participants with AD performed better on all assessments, with the exception of grip strength, compared with participants with vascular dementia and DLB.

This corroborates the results obtained in our study with a correlation of the MMSE with the different assessments of the gait parameters in DLB but remaining more discrete in AD with a correlation at the limit of significance only for the TUG.

### Gray matter, cognitive function and gait parameters

This study highlighted a difference in total GM volume between the groups. A decreased volume of GM in AD compared to DLB was expected [21].

There was also a correlation between total GM volume and cognitive assessment using the MMSE in the AD group and the DLB group, but not in the HEC group. This is in agreement with the study by Choi et al. [22], who showed a significant association between GM volumes and MMSE in AD patients with and without Lewy bodies.

Regarding gait parameters and total GM volume, there was a correlation only for gait speed in the HEC and DLB groups, but not in the AD group; however, there was no correlation between GM volume and TUG or single-leg balance in any of the groups.

Lee et al. have shown similar results in a population of healthy older adults [23] and one study examined GM volume in dementia with Lewy bodies and correlated it with double-task walking. In this study, the authors demonstrated that GM volume loss was associated with worse dual-task gait performance compared to single task gait, across cognitively unimpaired controls through and the Lewy body disease spectrum [24]. We found no previous studies showing a correlation between gait speed and GM volume in AD populations.

### VBM analysis in DLB group

To our knowledge, this is the first study to examine the correlation between walking speed or spatiotemporal gait parameters and GM volume loss in specific brain regions in the DLB population.

Regarding our results on the brain regions involved in reducing walking speed, the limbic region appears to be important. Some authors have found no significant difference in hippocampal volume between patients with AD-DLB and patients with “pure” DLB, even after correction for age and sex [25]. For other authors, DLB is more likely to be characterised by a spared hippocampus [26] but could nevertheless lead to medial temporal lobe atrophy [27]. Loss of hippocampal volume has been shown to lead to memory deficiency but it also appears to be linked to poor walking performance in people with mild cognitive impairment [10, 11]. This relationship between walking speed and the hippocampus could be linked to its involvement in spatial navigation and visual processing [28, 29].

We used the subdivisions of the cingulate cortex proposed by Vogt in 1993. He defined a subdivision into four structures: the ACC, the MCC, the posterior cingulate cortex and the retrosplenic cortex. The anterior and mid-cingulate cortices are part of the limbic system and form the interface between emotions (anterior cortex) and cognition (midcingulate cortex). These cortices integrate data from a variety of sources, including motivation, error evaluation, emotions and cognition. It has been suggested that the anterior part of the midcingulate cortex is used to modulate activity in the executive regions of the brain that direct attention and produce motor responses [30]. This function could therefore play a role in walking speed.

Our results suggest that the limbic system (cingulate cortex and hippocampi) is involved in gait disorders in DLB patients, underlining the existence of a motivational and emotional component responsible for slower walking speed. The correlations found with the hippocampus may be linked to its role in the topographical memory and mental navigation required for walking.

Other studies highlighted the involvement of the cingulate cortex and hippocampus in spatiotemporal gait parameters in healthy older populations. Rosso et al. showed an association between step length variability and GM integrity for the hippocampus and anterior cingulate gyrus as compared to other brain regions indicating a role for cognitive function in motor control [31]. Rosano et al. revealed that shorter steps and longer double support times were associated with smaller sensorimotor regions and also with smaller frontoparietal regions within the motor, visuospatial, and cognitive processing speed domains [32]. The DiSalvio study showed that greater parahippocampus GM volume was independently associated with greater gait speed [33]. Finally, in a population of older adults with amnestic mild cognitive impairment, McGough et al. found a moderate positive correlation between hippocampal and anterior cingulate volumes and gait and executive functions [34].

Caudate nuclei is divided into two parts according to its functions and connectivities : a dorsal part, involved in motor skills and cognitive functions [35] and implicated in spatial working memory [36], and a ventral part, which is more associated with the treatment of rewards, affects such as pain and even fatigue [37]. Allali et al. showed in older people without neurocognitive disorders that brain regions differed according to walking conditions and that three main individual GM regions were positively correlated with walking speed: right thalamus, right caudate nucleus, and left middle frontal gyrus for normal walking, rapid walking, and dual-task walking condition, respectively [38]. A correlation between attention and processing speed disorders and the caudate nuclei has also been demonstrated [39, 40]. Moreover using the same cohort as for this study, we have demonstrated that the decrease of cognitive speed processing in DLB was correlated with caudate nuclei [41]. This could mean that the caudate nuclei underlie overall brain processing speed, both cognitive and physical in DLB patients.

In the DLB group, there was a significant correlation with the cerebellum GM concentration probably due to its role in motor cognitive control, motor programming and motor coordination [42, 43]. We also found the involvement of the left parietal operculum, which persisted when we took laterality as a covariate. The parietal operculum is a structure located in the lower part of the parietal lobe, above the insula, in which the secondary somatosensory cortex is located [44]. This cortex is believed to be involved in pain perception, tactile attention, visceral sensations, but also, attention to the spatial location of an object and intentional motor behaviour, which may be involved in walking [44]. This cerebral region has, therefore, connections to the parietal perceptual network as well as to the motor frontal areas [45]. Finally, concerning the SMA, a link has been demonstrated in planning and execution of simple to complex motor tasks [46]. Some authors have also shown a functional connectivity between the MCC and motor frontal regions [47], particularly with the primary motor cortex and the SMA, making this cingulate region an area of intentional motor control [48], at a higher level than the SMA [49].

Walking disorders in patients with DLB are multifactorial. The extrapyramidal syndrome obviously plays a part in these disorders but does not appear to be the only factor involved. In fact, our study shows that these gait disorders, and particularly a slower walking speed, appear to be linked to a reduction in the volume of GM in structures involved in cognitive, spatial navigation, motivational, motor control with processing speed and balance, independently of cognitive fluctuations and the parkinsonism score.

### Limitations section

Several limitations need to be considered. This was a cross-sectional study, which did not allow us to assess the temporal direction of the associations. However, our analysis benefited from the fact that it was conducted in a well-characterized cohort with clearly established diagnoses, and that the DLB cohort was compared with the other two groups of patients. This enabled us to adjust for several potential confounding factors.

There is probably a lack of power in the HEC group, given the limited number of subjects. For the AD group, we cannot exclude the same problem. However, given the limited number of articles published on this subject, the number of participants to be included was impossible to calculate in advance. On the other hand, with almost 40 participants presenting with AD, and given the significant cerebral atrophy visualized on MRI if there were significant differences they should have been highlighted. Furthermore, similar studies on other characteristics have shown correlations with an equivalent or even smaller number of participants [50–53].

It is difficult to obtain accurate data on the number of falls in this older population suffering from neurocognitive disorders, as there is a reporting bias with a risk of forgetting the number of falls and a willingness to underestimate it. However, it was possible to determine the profile of patients who fall (occasional or recurrent) fairly easily.

We studied gait disorders only in association with GM density. Other brain imaging parameters should be evaluated to better understand the neural correlates associated with gait speed in the DLB population (perfusion with arterial spin labeling, functional connectivity with functional MRI, anatomical connectivity with diffusion tensor imaging).

## Conclusion

We describe here for the first time the neural basis of walking speed in DLB. Indeed, there is a motivational and emotional component in voluntary walking in subjects with DLB, underpinned by the cingulate cortex, a spatial orientation component, underpinned by hippocampi, as well as the involvement of cerebral processing speed and extrapyramidal circuit, underpinned by the caudate nuclei. This work should be completed by a study of the anatomical and functional connectivity between all these structures in order to identify brain networks responsible for gait disorders in DLB.

## Data Availability

The datasets used and/or analysed during the current study are available from the corresponding author on reasonable request.

## Abbreviations

ACC: Anterior Cingulate Cortex
AD: Alzheimer’s Disease
ADL: Activities of Daily Living
ANOVA: Analysis Of Variance
CM2R: Memory Resource and Research Center
DARTEL: Diffeomorphic Anatomical Registration Through Exponentiated Lie algebra
DLB: Dementia with Lewy bodies
FDR: False Discovery Rates
FWE: Family-Wise Error GM: Gray Matter
HEC: Healthy Elderly Controls
IADL: Instrumental Activities of Daily Living
MCC: MidCingulate Cortex
MMSE: Mini-Mental State Examination
MNI: Montreal Neurological Institute
MRI: Magnetic Resonance Imaging
PO: Parietal Operculum
SD: Standard Deviation
SMA: Supplementary Motor Area
SPM12: Statistical Parametric Mapping 12
TUG: Timed Up and Go test
UPDRS III: Unified Parkinson’s Disease Rating Scale III
VBM: Voxel-Based-Morphometry
